# Antibiotics Modulate Intestinal Regeneration

**DOI:** 10.3390/biology10030236

**Published:** 2021-03-19

**Authors:** Lymarie M. Díaz-Díaz, Natalia Rosario-Meléndez, Andrea Rodríguez-Villafañe, Yariel Y. Figueroa-Vega, Omar A. Pérez-Villafañe, Angela M. Colón-Cruz, Paola I. Rodríguez-Sánchez, Julio M. Cuevas-Cruz, Sonya J. Malavez-Cajigas, Sergio M. Maldonado-Chaar, José E. García-Arrarás

**Affiliations:** Río Piedras Campus, University of Puerto Rico, San Juan, PR 00931-3360, USA; lymarie.diaz@upr.edu (L.M.D.-D.); rosariomelnd@wisc.edu (N.R.-M.); andrea.rodriguez32@upr.edu (A.R.-V.); yariel.figueroa@upr.edu (Y.Y.F.-V.); omar.perez17@upr.edu (O.A.P.-V.); acolon20@stu.psm.edu (A.M.C.-C.); paola.rodriguez27@upr.edu (P.I.R.-S.); juliocc@sanjuanbautista.edu (J.M.C.-C.); sonya.malavez@upr.edu (S.J.M.-C.); smaldonado19@stu.psm.edu (S.M.M.-C.)

**Keywords:** antibiotics, intestinal regeneration, sea cucumber, *Holothuria glaberrima*, gut microbiota, toxicity

## Abstract

**Simple Summary:**

The impact of the microbial community on host’s biological functions has uncovered the potential outcomes of antibiotics on host physiology, introducing the caveats of the antibiotic usage. Within animals, the digestive function is closely related to the microorganisms that inhabit this organ. The proper maintenance of the digestive system requires constant regeneration. These processes vary from self-renewal of some cells or tissues in some species to the complete regeneration of the organ in others. Whether antibiotics influence digestive organ regeneration remains unknown. We employ the sea cucumber, *Holothuria glaberrima*, for its capacity to regenerate the whole intestine after ejection from its internal cavity. We explored the antibiotics’ effects on several intestinal regeneration processes. In parallel, we studied the effect of antibiotics on the animals’ survival, toxicity, and gut bacteria growth. Our results show that tested antibiotics perturbed key cellular processes that occur during intestinal regeneration. Moreover, this happens at doses that inhibited bacteria growth but did not alter holothurian’s metabolic activity. We propose that antibiotics can perturb the cellular events of intestinal regeneration via their impact on the microbiota. These results highlight *H. glaberrima* as a promising model to study the importance of the microbiota during organ regeneration.

**Abstract:**

The increased antibiotics usage in biomedical and agricultural settings has been well documented. Antibiotics have now been shown to exert effects outside their purposive use, including effects on physiological and developmental processes. We explored the effect of various antibiotics on intestinal regeneration in the sea cucumber *Holothuria glaberrima*. For this, holothurians were eviscerated and left to regenerate for 10 days in seawater with different penicillin/streptomycin-based cocktails (100 µg/mL PS) including: 100 µg/mL kanamycin (KPS), 5 µg/mL vancomycin (VPS), and 4 µg/mL (E4PS) or 20 µg/mL (E20PS) erythromycin. Immunohistological and histochemical analyses were performed to analyze regenerative processes, including rudiment size, extracellular matrix (ECM) remodeling, cell proliferation, and muscle dedifferentiation. A reduction in muscle dedifferentiation was observed in all antibiotic-treated animals. ECM remodeling was decreased by VPS, E4PS, and E20PS treatments. In addition, organisms subjected to E20PS displayed a significant reduction in the size of their regenerating rudiments while VPS exposure altered cell proliferation. MTT assays were used to discard the possibility that the antibiotics directly affect holothurian metabolic activity while bacterial cultures were used to test antibiotic effects on holothurian enteric microbiota. Our results demonstrate a negative effect on intestinal regeneration and strongly suggest that these effects are due to alterations in the microbial community.

## 1. Introduction

The use of antibiotics has increased drastically over the last decades. Their wide use in human and veterinary medicine has been attributed to their many beneficial effects including the prevention and treatment of pathogen-associated diseases. Moreover, the use of antibiotics has extended to other farming activities, including agriculture and aquaculture [1,2] to improve the culture conditions for mass production and to maintain sterile conditions for research purposes [3,4]. Antibiotics are not only used for traditional livestock or piscine species, but their use extends to many other types of agriculture/aquaculture, such as fish, shrimp, crab, oysters, and mussels [5,6,7,8,9,10,11,12,13,14,15,16,17,18,19,20,21].

Among the most used antibiotics worldwide are tetracycline, penicillin, sulfonamides, and macrolides. However, many other antibiotics are also known for their agriculture/aquaculture use. For example, quinolones, sulfonamides, tetracyclines, erythromycin, and oxytetracyclines are used to treat septicemia, skin diseases, and other bacterial infections in a variety of fish species [13,22,23]. The cocktail of sulfadiazine and trimethoprim, known as tribrissen, has also been employed to treat the vibriosis for flatfish, jacopever, yellowtail, and salmon [13,24]. Antibiotics have also been used for sea cucumber aquaculture where cultures of *Apostichopus japonicus* are treated to prevent diseases such as Skin Ulceration Syndrome caused by *Vibrio* sp. [2,25].

Moreover, antibiotics are also known to increase the survival or promote the growth of an organism. This phenomenon is well documented in vertebrates [23,26,27]. However, even in invertebrates, antibiotics have been shown to increase survival and act as growth promoters [28,29,30]. For example, insects cultured in the presence of antibiotics show accelerated growth [29] and pupation [31].

It is important to state that antibiotics can also have an adverse effect on cultured organisms and that the balance lies on the applied dosage. While it has been reported that streptomycin sulphate improves the development of some insects [32], adverse effects have been found when these are treated with higher concentrations [33]. In addition, a comprehensive study in two invertebrates (*Dapnia magna* and *Moina macrocopa*) and the medaka fish, found that chronic exposure for 21 days with “no-observed-effect concentrations” of antibiotics caused adverse effects on the survival and growth of larvae, as well as negative reproductive and hatching effects [34]. Also, antibiotics as rifampicin cause a decrease in the post-larval survival of *P. turionellae* [31].

Since oral administration is a common route of antibiotic treatment, the responses of pharmaceuticals on the digestive tract have been frequently studied. These outcomes can be directly acting on the organism’s cell functions and metabolism, as erythromycin, which acts as a motilin receptor agonist, causing a prokinetic action on the gut [35], or can be indirect via their antimicrobial actions and the subsequent changes in microbiota. For instance, the prolonged use of antibiotics on mammals decreases the diversity and causes the translocation of commensal gut bacteria, and induces inflammatory responses [36] which alters the hosts’ cell renewal capacity.

Furthermore, many antibiotics prescribed for infections can target the commensal microbiota and promote gut dysbiosis [37,38,39,40,41,42,43,44,45], diminished short-chain fatty acids (SCFAs) [39,41,46,47], morphological changes to the gastrointestinal tract (GIT) villi [48], intestinal mucosa damage [49], intestinal permeability [39], and pathogen infiltration [42,43,44,49]. Those antibiotic-associated alterations perturb gut homeostasis, leading or perpetuating intestinal diseases. Such diseases include small intestine bowel overgrowth (SIBO) [50], gastroenteritis [38], inflammatory bowel disease (IBD) [38,49,51], *Clostridium difficile* disease [52], colitis [45,53,54], colorectal cancer [51,55], among others.

Even though there are numerous studies about the effect of antibiotics on the digestive tract, the impact of antibiotics on the intestinal regeneration has been limited to the regeneration or healing of the mucosal epithelium. Many studies in germ-free models revealed the influence of the association between the mucosa and the microbiota, which can be altered by antibiotics, in the maintenance of the enteric system [56]. Accordingly, the function of enteric glial cells is important for the intestinal barrier integrity [57] and prevention of Crohn’s disease [58] and colitis [59] through expression of Toll-like receptors (TLRs). Moreover, another study showed that antibiotic treatments can hinder the intestinal TLR signaling response to LPS, altering the production of colony-stimulating factor 1 (CSF-1), leading to severe effects on peristalsis [60]. An impaired mucosal barrier and incorrect immune activation by commensals mislocalized to the mucosa are associated with IBD occurrence [51,61,62]. IBDs can be perpetuated after antibiotic exposure if the microbes localized on the crypts and transverse folds recedes the colon during digestion [63].

Notwithstanding, the luminal epithelium is only one of the cellular layers of digestive tract organs. Thus, the question of how antibiotics affect organ regeneration, remains unanswered, or at best, only partially resolved. Using our model system, the sea cucumber *Holothuria glaberrima*, we now have the opportunity to answer this question. These organisms regenerate their complete intestine, providing a unique animal model to study organ regeneration [64]. Our laboratory has extensively studied the cellular and molecular processes that underlie the process of intestinal regeneration [65,66]. In brief, following evisceration, regeneration of the lost organ begins by wound healing of the anterior and posterior free ends of the gut (adjacent to the esophagus and cloaca, respectively) and a significant reorganization of the mesentery [67]. The 7-dpe rudiment, which forms at the tip of the free end of the mesentery, is characterized by concurrent processes that begin after wound healing and continue until the lumen is formed. One of these processes is the dedifferentiation of the mesenterial muscle layer, in which dedifferentiated myoepithelial cells condense their actin filaments into SLS [68,69]. This rearrangement of the cytoskeleton changes the shape of the dedifferentiated mesothelial cells [70], which possibly results in migration to form the rudiment. Another process is the reorganization of the ECM, characterized by collagen fiber disappearance from the connective tissue by the action of distinct proteases, such as matrix metalloproteinases [71]. The third process that takes place during the first week of regeneration is cell division. From 5-dpe to 10-dpe after evisceration, significant higher levels of cell proliferation are found in the mesothelium of regenerating rudiments. At 7-dpe, cell division increases in the coelomic epithelium, and, to a lesser extent in the connective tissue [67]. After a week, a blastema-like structure can be found along the free edge of the mesentery. This rudiment is connected at the anterior end with the remnants of the esophagus, and at the posterior end with the cloaca. 

Moreover, sea cucumbers are deuterostomes, thus, their phylogenetic relationship to vertebrates fosters the use of this organism as a key model for understanding regeneration in vertebrates, including humans.

Among the echinoderms, the effects of antibiotics on regeneration have only been studied in *Apostichopus japonicus* sea cucumbers. However, these studies have been focused on their effects on weight and survival because of their commercial value for Asian cultures. We now explore the effect of antibiotics on the intestinal regeneration process and, in parallel, their effect on holothurian tissues and enteric bacteria. For this, penicillin and streptomycin-based cocktails, kanamycin, vancomycin, and erythromycin were administered to sea cucumbers undergoing regeneration of their digestive tract. In addition, we examined the toxicity to holothurian cells and explants, the antibiotics’ minimum inhibitory concentration (MIC) over bacteria isolated from sea cucumber gut detritus, and the survival rate of sea cucumbers exposed to antibiotics. Our results show that penicillin and streptomycin-based cocktails perturbed the ECM remodeling, cell dedifferentiation, and cell proliferation processes that lead to gut formation. Moreover, the antibiotic effect on the intestinal regeneration process takes place at doses that inhibit gut bacteria growth but do not alter the holothurian cell metabolic activity, suggesting that the observed effect on organ regeneration might be triggered by changes in the microbiota.

## 2. Materials and Methods

### 2.1. Antibiotic Effect on Intestinal Regeneration

#### 2.1.1. Animal Care

Adult sea cucumbers were collected on the northeast coast of Puerto Rico. They underwent an acclimatization process to adapt them to new conditions. For this, the animals were placed in aquaria and exposed to three days of water change; the first with filtered natural seawater (FNSW), then to 50:50 FNSW and filtered artificial seawater (FASW), and lastly, 100% FASW (37 g/L Reef Science Instant Ocean Water). After acclimatization, evisceration was induced using 0.35 M KCl (3–5 mL per animal) and animals were transferred to new autoclaved aquaria. Each aquarium contained 1 L FASW, and a maximum of two animals. Penicillin/streptomycin (10,000 units penicillin and 10 mg streptomycin per mL in 0.9% NaCl, sterile-filtered, BioReagent, P078, Sigma-Aldrich^R^, St. Louis, MO, USA), vancomycin hydrochloride (Fisher BioReagents^TM^,Thermo Fisher Scientific, Waltham, MA, USA), kanamycin sulfate (Sigma-Aldrich^R^, St. Louis, MO, USA), and erythromycin (Sigma-Aldrich^R^, St. Louis, MO, USA) were used. Penicillin-streptomycin to a final concentration (FC) of 100 µg/mL (PS) was added to all experimental groups (Table S1). A basic dose of PS served to target both Gram-negative and Gram-positive bacteria as well as to avoid pathogen-associated diseases. PS-based cocktails were additionally supplemented with kanamycin (FC = 100 µg/mL; KPS), vancomycin (FC = 5 µg/mL; VPS), or erythromycin (FC = 4 µg/mL; E4PS or 20 µg/mL; E20PS, respectively. Additional supplementation of kanamycin was added to select against remaining or PS-resistant Gram −, while erythromycin and vancomycin were added to select against remaining or PS-resistant Gram +. Every two days the water was changed, and the drugs were re-administered likewise. Non-treated (SW) controls were transferred to FASW; no drug was administered to these animals, but they also underwent water changes (Table S1).

On day 9 post evisceration (dpe), animals were injected with 0.5 mg of BrdU (SIGMA, Cat. #B5002, St. Louis, MO, USA) per g of animal weight. At 10-dpe, 12 h after injection, organisms were anesthetized in 0.2% 1, 1, 1-trichloro-2-methyl-2-propanol hydrate sedative solution in seawater, for at least 30 min, and sacrificed. The medial section of the regenerating intestine of each organism was dissected and fixed overnight with 4% paraformaldehyde in 0.1 M PBS at 4 °C. Afterwards, tissues were rinsed with 0.1 M PBS for 15 min, three times, and then left in 40% sucrose and stored at 4 °C, for tissue preservation. Tissues were embedded in OCT Compound Tissue-Tek, cryosectioned at 20 μm in a Leica CM1850 cryostat and mounted onto slides treated with poly-L-lysine. At least, nine animals were used per treatment.

#### 2.1.2. Immunohistochemistry

Several cellular processes involved in intestinal regeneration were evaluated for potential effects of the antibiotic treatments. The protocols for immunohistochemistry performed in our laboratory have been described previously [67,72,73]. In brief, approximately 50 μL of primary anti-collagen (E6D9G3) antibody was applied to sections and incubated overnight in a humid chamber at room temperature. The next day, slides were rinsed with 0.1 M PBS three times for 15 min each. In some cases, slides were treated with 1/50 goat serum before the application of the primary antibody, to decrease nonspecific background fluorescence. Slides were then incubated with GAM-CY3 (BioSource Int., Camarillo, CA, USA) secondary antibody, for 1 h, and washed again three times for 15 min each, with the same buffer. Muscle labeling was done by adding fluorescent-labeled phalloidin during the incubation with the secondary antibody antibody as published previously [67]. Phalloidin-TRITC (Sigma P1951, St. Louis, MO, USA) was used at a final concentration of 1:2500. Slides were mounted in a buffered glycerol solution containing 1 µg/mL of 4′,6-diamidino-2-phenylindole (DAPI, Sigma, St. Louis, MO, USA), after three additional washes with PBS. The slides were analyzed under a Nikon Eclipse Ni fluorescence microscope and the Nikon DS-Qi2 camera was used to obtain images of regenerating rudiments.

#### 2.1.3. Measurement of Rudiment Area

Measurements of the rudiment area, as well as cell count, were done using ImageJ software (http://rsbweb.nih.gov/ij/ (accessed on 25 May 2018)). The area of rudiment was measured from at least nine animals per treatment (three sections each). To normalize measurements from different replicates, the average size of each animal regenerate was divided by the average of all PS (mean of individual averages) and represented as percent of size change. All values are reported as mean ± standard deviation.

#### 2.1.4. Remodeling of the ECM

ECM remodeling was studied by determining the loss of collagen from the regenerating tissues [71]. The level of ECM remodeling was classified using the scheme shown in Scheme S1A. In brief, this model adjudicates a value to the presence of collagen fibers along the length of the mesentery, from the body wall to the free margin where the intestinal rudiment is forming; a 0 corresponds to a rudiment that still retains a large amount of collagen, while a 5 corresponds to fibers being only present in the mesentery adjacent to the body wall, or not detectable at all. At least three animals were used for each experimental condition and at least two sections from each animal were counted and averaged per animal. All values are reported as mean ± standard deviation.

#### 2.1.5. Muscle Dedifferentiation

Muscle dedifferentiation was determined by the presence of spindle-like structures (SLSs) [68,74]. These structures contain contractile material from dedifferentiated cells. SLSs were detected using rhodamine-labelled phalloidin, as described previously, using the scheme shown in Scheme S1B, adjudicating a 0 to the presence of SLS or muscle fiber from the distal tip of the rudiment, and a 5 when the SLS and fibers were only visible in the mesentery adjacent to the body wall [75]. At least three animals were used for each experimental condition and at least two sections from each animal were counted and averaged per animal, values are reported as mean ± standard deviation.

#### 2.1.6. Cellular Proliferation

Cell division was determined using BrdU incorporation [65]. To detect cells that incorporated BrdU, slides were treated with Triton 100X (0.2%) for 15 min, two washes with 0.1 M PBS for 15 min, followed by one-hour (1 h) treatment with 0.05 M HCl, and another wash with PBS. Then the primary antibody murine monoclonal anti-5-bromodeoxyuridine (GE Healthcare Code: RPN 202) diluted 1:4 in RIA buffer, was applied to the sections and left overnight. Followed by the three washes of PBS, the incubation with the secondary antibody, the slides were mounted as described previously.

BrdU immunoreactive cells and DAPI labelled cells were counted, from at least three animals in each experimental condition and at least two sections from each animal. All the epithelial cells, as the cells in the connective tissue in the rudiment were counted, separately. The delimitation of these areas is presented in Scheme S2. The number of proliferating cells was normalized to the total number of cells labeled with DAPI within the visual field. Cell proliferation ratio (BrdU/DAPI labeled cells) was used to compare the different treatments. The values are reported as mean ± standard error of the mean (SEM).

### 2.2. Minimum Inhibitory Concentration (MIC)

#### MIC Determination

Sea cucumbers collected from the northeast coast of Puerto Rico were transferred to an aquarium with filtered artificial sea water (FASW) upon arrival at the laboratory. After 1 h, released gut detritus was collected and stored in 50% glycerol at −80 °C. For MIC determination, a first inoculum was performed by streaking the stored samples to a plate with Marine Agar (DifcoTM, Thermo Fisher Scientific, Waltham, MA, USA) with no selection media, and left for 2 d at 25 °C. Then, isolated colonies were inoculated separately into 5 mL of Marine Broth (Difco^TM^, Thermo Fisher Scientific, Waltham, MA, USA), for 5 to 18 h. 10^3^ bacteria were spread into agar plates with different concentrations (from 325 μg/mL to 0.5 μg/mL) of the antibiotics penicillin/streptomycin (PS), kanamycin (K), vancomycin (V), or erythromycin (E). Two negative controls were used: (1) plates were spread with sterile marine broth and (2) non-inoculated plates (blank). All Petri dishes were prepared with a total of 20 mL of marine agar per plate. For plates with selection media, the amount of drug needed to achieve each concentration was determined in a final volume of 20 mL of agar. At least four plates replicates were tested for each antibiotic dose. Bacteria growth was observed after plate incubation for 2 d at 25 °C.

### 2.3. Antibiotic Toxicity

Toxic effects of antibiotics on holothurian tissues were studied in two different preparations: muscle explants and isolated muscle cells (Supplemental Materials). For this we utilize the longitudinal muscle because it provides a large amount of tissue from an individual animal. In addition, a large component of the intestine is the muscle layer found in the mesothelium. This muscle has similar properties to the longitudinal muscle used in our assays.

#### 2.3.1. Muscle Dissection for In Vivo and Ex Vivo Assays

Muscle tissues were obtained following the dissection protocol described by Bello, SA, et al. 2015 [76], with some modifications. Briefly, animals were anesthetized by placing them on ice for 1 h, then washed with 10% sodium hypochlorite, ethanol 70% respectively for one minute, and left in purified/autoclaved sea water. Animals were dissected, and longitudinal muscles were carefully removed. Collected tissues were transferred to a 3X penicillin/streptomycin, neomycin, and amphotericin B antibiotic (3X abx) cold media. Upon removal, longitudinal muscles were transferred to 3X abx and left in a shaker for 1 h at room temperature. 

#### 2.3.2. Explant Culture and Toxicity Essay

The antibiotic effects on holothurian tissues were studied using longitudinal muscle explants. After 1 h in 3X antibiotic solution, muscle explant were cut to 3 mm diameter using a surgical punch and transferred to seawater supplemented media (3 g/L glucose, 2.86 g/L HEPES buffer, 1X penicillin/streptomycin, 50 ug/mL gentamicin, 1 mM sodium pyruvate, 1% MEM non-essential amino acids, 1.75 ug/mL tocopherol, and 2.5 ug/mL amphotericin B in FASW). Later, each punch-explant was transferred into one well and antibiotics were added to a final volume of 200 µL. Triplicates were prepared for each dilution. 

PS, K, E, and V were tested individually. PS and K doses included 10, 20, 30, 50, 125, 250, and 500, E and V doses included 2, 4, 6, 10, 25, 50, and 100 μg/mL. For cocktail assays, PS at a constant final concentration of 100 µg/mL was added to all other antibiotics’ dilutions. In addition to the PS similar doses of K, E, and V but doses of 100 μg/mL K, 20 μg/mL E, and 5 μg/mL V were also included.

Plates were incubated for 72 h at 25 °C. Later, the TOX1 Sigma In Vitro Toxicology Assay Kit (MTT based Sigma Aldrich, St. Louis, MO, USA) was used to determine the metabolic activity of sea cucumber explants exposed to antibiotics. Similarly to Nicol MR, et al., 2015 explant MTT assay [77], 200 µL of MTT solution (MTT reconstituted in culture media) were added to each well and incubated for 1 h following the kit’s protocol [78]. After incubation, each explant and supernatant were transferred to 500 µL of methanol, to extract all the formed formazan. This was incubated for a period of 36 h, in a gyratory plate to enhance dissolution. Following this procedure, 200 µL of the supernatant were transferred to a 96-well plate, their 570 nm absorbance was recorded, and background absorbance (690 nm) subtracted. 

Net MTT absorbance:

OD_597nm_ − OD_650nm_, (1)

where OD_597nm_ is used to detect the formazan and OD_650nm_ detects the background noise.

#### 2.3.3. Protein Quantification for MTT Normalization

To improve the quantification values, explants were lysed after the MTT assay to measure the protein quantities and these were used to determine the metabolic activity as a value of protein amount. For this, 1200 μL 1X RIPA (lysis buffer) was added to each tissue explant, for ultrasonic lysis using a Branson Fisher Scientific 150E Sonic Dismembrator model 150E for 60–90 s at an ultrasonic cycle mode of 25 s sonication and, at least 25 s resting time in ice. After ultrasonic homogenization, the lysate was centrifuged at 27,000 rcf for 20 min. The supernatant was collected, and the Pierce protein assay BCA kit (Thermo Scientific™, Waltham, MA, USA) was used to determine protein concentration. Bovine Serum Albumin was used as standard. About 25 μL of each standard or unknown sample replicate, in addition to 200 μL of BCA Working Reagent (WR), was pipetted into each microplate well and mixed thoroughly. Plates were covered and incubated at 37 °C for 30 min. The absorbance was measured at 562 nm using the plate reader SpectraMax 360, and a customized protocol to record those absorbances using SoftMax Soft. GraphPad Prism 6.0 was used to interpolate values from BSA standardization to relative protein concentration. 

Metabolic activity rate in explants were obtained by dividing the measure of metabolic activity (absorbance) by the relative protein concentration from each sample.

Explants normalized absorbance:

(2) $$\frac{\mathrm{net}\;\mathrm{MTT}\;\mathrm{absorbance}}{{\mathrm{OD}}_{562\;\mathrm{nm}}\;}\times 100,$$

where OD_562nm_ is used to detect protein concentration in each explant.

Metabolic activity rate in explants:

(3) $$\frac{\mathrm{treated}\;\mathrm{explants}\;\mathrm{normalized}\;\mathrm{absorbance}}{\mathrm{nontreated}\;\mathrm{explants}\;\mathrm{normalized}\;\mathrm{absorbance}}\times 100,$$

where treated and nontreated normalized absorbance are the normalized absorbance of each explant treated with antibiotics and non-treated explants, respectively. Mean results where graphed using PRISM GraphPad 6.0, and error bars show the SEM for each treatment.

Results were graphed as mean ± SEM. A non-linear regression curve fit [log(inhibitor or agonist) vs. response—Variable slope] was done to determine the IC50 or EC50 of these doses using GraphPad PRISM. A Spearman correlation test was performed, in addition to Wilcoxon *t*-test and Kruskal-Wallis (ANOVA) to evaluate dose-dependent effects compared to non-treated cultures.

### 2.4. Statistical Analyses

Statistical analyses were performed using Mann–Whitney (MW) *t*-test to compare each group against the appropriate controls, the Krustal–Wallis test was also used to compare all the groups, for in vivo experiments. A Wilcoxon *t*-test analysis as well as Krustal–Wallis (KW) test was performed to analyze toxicity assays. Results are reported as mean ± SD or SEM (specified in figures legends). Statistical significance is represented with asterisks (*) and *p*-value (*p*).

## 3. Results

### 3.1. Survival Rate in Regenerating Sea Cucumbers during Antibiotic Treatments

To determine whether the antibiotic treatments influenced sea cucumber viability, a survival analysis was performed. A total of 76 animals were eviscerated and treated with one of the following antibiotic treatments: SW n = 14, PS n = 19, KPS n = 12, E20PS n = 12, E4PS n = 9, VPS n = 10. All animals treated with KPS, E4PS, and VPS survived the 10-dpe treatment (Figure S1). For other treatments, survival rates varied from 83% (E20S) to 91% (untreated controls). However, a comparison of survival curves was done using a Log-rank (Mantel-Cox) test and the curves did not appear to be statistically different (*p* = 0.4774) (Figure S1).

### 3.2. Rudiment Formation Is Perturbed by Erythromycin Exposure

To test whether exposure to antibiotics alters the intestinal regenerative process of the sea cucumber *H. glaberrima*, animals were subjected to various antibiotics for 10-dpe following evisceration. Several parameters associated with intestinal regeneration were assessed, including the size of the regenerating gut and the extent of ECM remodeling, cell dedifferentiation, and cell proliferation.

**Figure 1:**
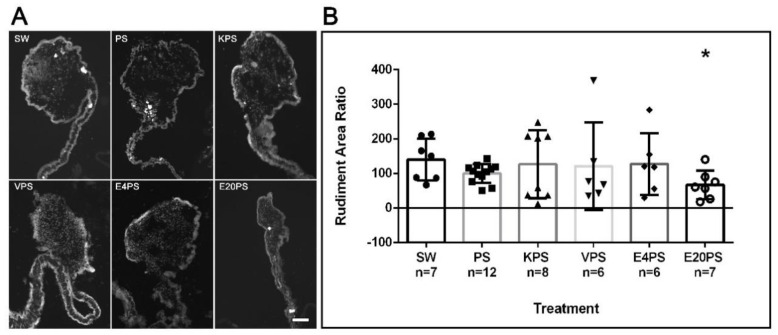
Rudiment growth after antibiotics exposure for 10-dpe. Sections of regenerating intestines from animals subjected to various antibiotic treatments including untreated controls (SW), and penicillin/streptomycin (PS) alone as well as kanamycin (KPS), vancomycin (VPS), and erythromycin (E4PS and E20PS) based cocktails-treated animals (**A**). Scale bar represents 100 μm. Percentage of the rudiments’ area in comparison with the PS group (**B**). Blastema size was measured using the program ImageJ. Bars show the mean of at least six (6) animals, ±SD graphed using GraphPad PRISM. Asterisk shows the result of *t*-test comparisons between SW and E20PS (* *p* < 0.05).

To evaluate whether the antibiotics perturbed the formation of the gut rudiment during the regeneration process, the rudiment area of antibiotic-treated organisms and controls was measured. PS-treated animals, as well as KPS, E4PS, and VPS treated animals showed similar size rudiments when compared to non-treated controls (Figure 1). However, the E20PS-treated group showed a smaller rudiment compared to PS or non-treated organisms (Mann–Whitney *t*-test *p* = 0.0262). The effect of erythromycin was only observed at the higher dose since animals treated with erythromycin at the lower concentration of 4 µg/mL (E4PS) did not show a significant decrease in the regenerating rudiment’s area (Figure 1). These results suggest that higher concentrations of erythromycin decrease the size of the intestinal rudiment of regenerating animals.

### 3.3. Intestinal Cellular Dedifferentiation Is Delayed by Antibacterial Treatments

The dedifferentiation of the muscle layer of the mesentery is one of the first processes observed following the evisceration process. Specifically, myoepithelial cells condense (pack) their actin filaments into spindle-like structures (SLS) [69]. This process occurs in a gradient, starting at the free end of the mesentery and moving toward the end attached to the body wall.

**Figure 2:**
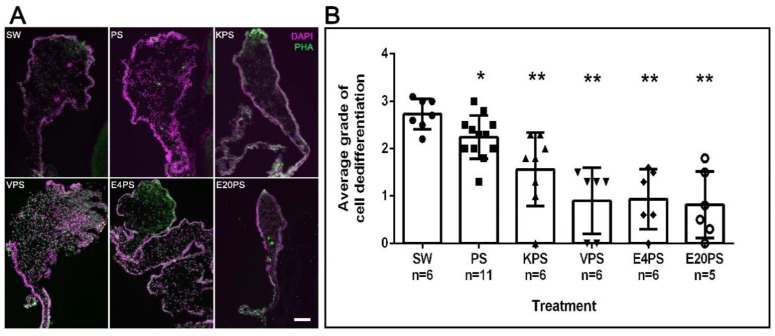
Muscle dedifferentiation in antibiotic treated regenerating guts. Muscle dedifferentiation was detected by the presence of SLSs and muscle fibers, labeled with Phalloidin-TRITC (green), and nuclei, labeled with DAPI (magenta) in regenerating guts of animals treated with PS, KPS, VPS, E4PS, and E20PS (**A**). Scale bar represents 100 μm. Dedifferentiation grade was determined using the algorithm shown Scheme S1B (**B**). Bars represents the mean of at least five (5) animals, ±SD. Asterisks show *t*-test comparisons between SW and experimental groups * *p* < 0.05, ** *p* < 0.01. Results were graphed using GraphPad PRISM.

As expected, in rudiments from 10-dpe non-treated animals SLSs and muscle fibers had already been eliminated from the area close to the forming rudiment but were observed in the mid region of the mesentery and close to the body wall (Figure 2B). Interestingly, all treated groups show differences compared to the control. However, each group varies from the least dramatic difference (PS treated) where the density of SLS was observed adjacent to the rudiment, followed by animals treated with kanamycin (KPS), that exhibited SLSs in or adjacent to the rudiment. In contrast, the rudiments from animals treated with vancomycin (VPS) and erythromycin (E4PS and E20PS) presented the most dramatic differences, characterized by the presence of SLSs and muscle fibers adjacent to the rudiment (Figure 2A). The Mann–Whitney t-test analysis suggested that all treated animals significantly differed from the non-treated controls (VPS vs. SW *p* = 0.0012, VPS vs. PS *p* = 0.0004, E4PS vs. SW *p* = 0.0012, E4PS vs. PS *p* = 0.0004, E20PS vs. SW *p* = 0.0012, E20PS vs. PS *p* = 0.0004, KPS vs. SW *p* = 0.0011, KPS vs. PS *p* = 0.0225 PS vs. SW *p* = 0.0201) as shown in Figure 2B. In addition, the non-parametric test of Gaussian distribution Kruskal–Willis test showed an approximate *p* = 0.0022, suggesting a perturbed cell dedifferentiation in animals treated with antibiotics.

### 3.4. ECM Remodeling Is Altered by Antibiotics

**Figure 3:**
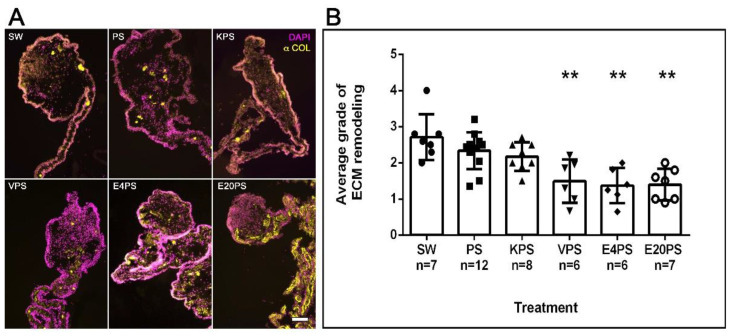
ECM remodeling after antibiotic treatments. Collagen presence was determined using the antibody E6D9G3. Non-treated animals (SW), animals treated with PS and KPS, VPS, E4PS, and E20PS were labeled with anti-collagen (yellow) and DAPI staining (magenta) (**A**). Scale bar represents 100 μm. Remodeling grade was determined using the algorithm shown in Scheme S1A (**B**). Bars represent the mean of at least six (6) animals, ±SD. Asterisks show t-test comparisons between SW and experimental groups ** *p* < 0.01. Results were graphed using GraphPad PRISM.

A significant remodeling of the ECM takes place during intestinal regeneration, which can be followed by observing the disappearance of collagen fibers in a graded process that begins near the forming rudiment and moves toward the mesenterial end attached to the body wall [71]. The effect of antibiotics on ECM remodeling process during regeneration was determined by labeling collagen expression. As expected, in SW controls, this ECM component had disappeared from the rudiment and was observed from the mid part mesentery to the body wall (Figure 3A). However, while some PS- or KPS-treated animals displayed this pattern, others showed the presence of collagen in the mesentery region adjacent to the rudiment. Moreover, all E4PS, E20PS, and VPS treated animals displayed collagen fibers in, or adjacent to, the rudiment suggesting a lag in the ECM degradation. To quantify these results, the algorithm shown in Scheme S1A was employed to classify ECM remodeling (via collagen presence) in the treated and untreated animals. SW controls, PS-treated, and KPS presented an average dedifferentiation grade of 2.7, 2.3 and 2.2, respectively. However, in both E20PS and E4PS the average value was 1.4 and 1.5 for the VPS-treated group (Figure 3B). Our statistical analysis (MW *t*-tests) reports that both the animals treated with vancomycin or erythromycin have significant collagen fibers in or adjacent to the rudiment compared with control groups (VPS vs. PS *p* = 0.0044, VPS vs. SW *p* = 0.0023, E4PS vs. PS *p* = 0.0018, E4PS vs. SW *p* = 0.0017, E20PS vs. PS *p* = 0.0020, and E20PS vs. SW *p* = 0.0012). In addition, the Kruskal–Willis test showed a *p* < 0.0005 which suggests that both vancomycin and erythromycin hinder the process of ECM remodeling.

### 3.5. Vancomycin PS-Based Treatment Alters the Cell Proliferation Rate in the Connective Tissue

**Figure 4:**
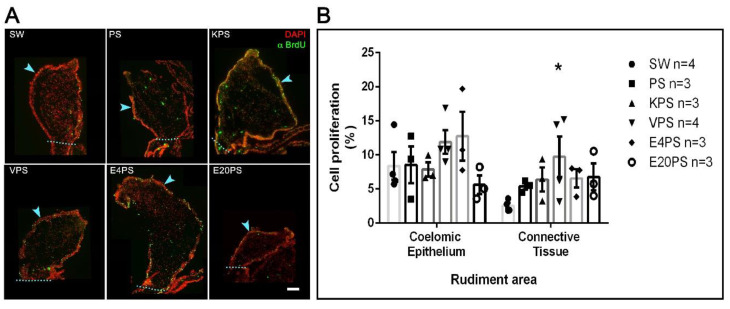
Antibiotic treatment effects on cellular proliferation. BrdU+ cells and DAPI labeled nuclei in the rudiment. Non-treated animals (SW), animals treated with PS and KPS, E20PS, E4PS, and VPS were labeled with anti-BrdU (green) and DAPI staining (red) (**A**). The light blue arrowheads point the coelomic epithelium and the dashed line delimits the rudiment area where cells were counted. Scale bar represents 100 μm. Cell division rate was calculated in coelomic epithelium and connective tissue by dividing the number of proliferative cells (BrdU+ cells) per total cells (DAPI+ nuclei) (**B**). Bars represent mean values of at least three (3) animals and SEM are displayed in the error bars. Asterisk shows *t*-test comparisons between the connective tissue of SW and experimental groups * *p* < 0.05. Results were graphed using GraphPad PRISM.

Our results show a 9% average proliferation in the coelomic epithelium and 3% in the connective tissue of the SW group animals (Figure 4). Thus, as expected for the 10-dpe, there is less cellular proliferation in the connective tissue when compared to the coelomic epithelium [67].

The average cell division ratio in the rudiment coelomic epithelium was approximately 10% in all treated groups (PS 9%, KPS 8%, VPS 12%, E4PS 13%, and E20PS 6%) with no significant differences when compared to controls (Figure 4). In the connective tissue of rudiments of animals treated with PS, KPS, E20PS, and E4PS, we observed an average cell division ratio of 5%, 6%, 7%, and 7% respectively, with no significant difference when compared to control groups. However, VPS rudiments showed a mean proliferation ratio of 10% and increased cell division in the connective tissue when compared to the rudiments of untreated animals (KW test *p* = 0.0429) (Figure 4).

A further statistical analysis comparing the proliferation rate in between the connective tissue and the mesothelium of each group showed that decreased cellular proliferation ratio in the connective tissue was only significant in non-treated animals (KW test *p* = 0.0048), and not observed in the experimental groups. This result suggests that all antibiotics tested perturbed the characteristic pattern of cell proliferation in 10-dpe regeneration intestines. 

There are at least two possible explanations for the observed antibiotic effects. First, the antibiotics might be having a direct effect on the sea cucumber tissues that alters their physiological/metabolic processes. Alternatively, antibiotics might be having an indirect effect on the sea cucumber physiology by causing dysbiosis of the intestinal microbiota. We explored both possibilities by first, using an MTT assay to determine the possible toxic effect of antibiotics on holothurian tissues and second, by determining the antibiotic effect on holothurian microbe cultures. 

### 3.6. Antibiotics and Holothurian Cellular Toxicity

#### 3.6.1. Cell Cultures

To determine if antibiotics have a detrimental effect in animal tissues we performed MTT assays using a primary muscle cell culture where cells were treated with antibiotics for 48 or 72 h (Figure S2A). This MTT assay is used to quantify cellular metabolic activity based on the reduction of (3-(4,5-dimethylthiazol-2-yl)-2,5-diphenyltetrazolium bromide (MTT) yellow salt to formazan purple crystals by metabolic active cells [79,80,81].

At low doses, most antibiotics showed little, if any effect on isolated cells with the sole exception of cells treated with erythromycin that had a decreased activity compared to non-treated cells supported by a significant negative correlation was found in cells treated with erythromycin for 48 and 72 h, (Spearman test r value of −0.6788 (*p* = 0.0240) and −0.8545 (*p* = 0.0015) respectively). After a non-linear regression “log(inhibitor) vs. response -Variable slope” curve fit determines the IC50 for cells incubated with this antibiotic is 1.29 µg/mL and 7.41 µg/mL for 48 and 72 h, respectively. A Kruskal–Wallis test showed significant decreased activity in cells treated with doses from 25 µg/mL compared with non-treated controls, for both 48 and 72 h (*p* = 0.0011, and *p* < 0.0001, respectively) (Figure S2C). On the other hand, no dose-dependent correlation was found for PS and K treatments (Figure S2B,D). However, a Dunn’s multiple comparisons test indicate that kanamycin alone significantly enhances cell metabolism after 72 h when treated at 50 µg/mL, 250 µg/mL, and 500 µg/mL doses (Kruskal–Wallis tests *p* = 0.0496) (Figure S2B).

Addition of erythromycin-PS cocktails reflect a negative correlation of r = −0.92 (*p* 0.0017) and an IC50 2.52 µg/mL at 48 h (Figure S2F). Consequently, an inhibitory response of erythromycin doses, from 6 µg/mL or higher, was confirmed with a Dunn’s multiple comparisons test and Kruskal–Wallis assay (*p* = 0.0002). Meanwhile, when kanamycin cocktails were added, neither an inhibitory nor agonistic effect could be detected (Figure S2E).

#### 3.6.2. Explant Cultures

**Figure 5:**
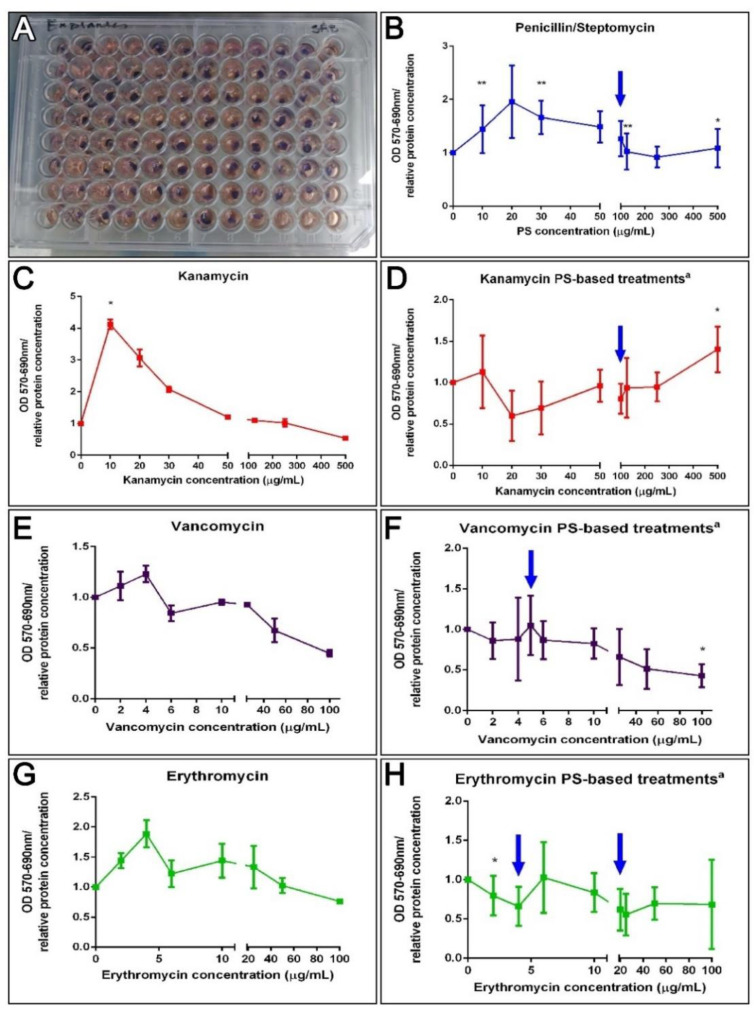
Ex vivo effect of antibiotics on the enzymatic activity of holothurians muscle explants. Results from ex vivo toxicity assay on sea cucumbers’ longitudinal muscle explants after incubation with antibiotics treatments for 72 h in culture, and 1 h in MTT (**A**). Dose effects after 72 h in culture with PS (**B**). The dose effects of kanamycin (K), vancomycin (V), or erythromycin (E) alone on holothurians muscle explants (**C**,**E**,**G**). On the right, are represented the dose effect explants in PS (100 μg/mL)-based cocktails, additionally supplemented with K, V, and E, respectively (**D**,**F**,**H**). Results are shown as the rate of metabolic activity normalized to the tissue density (MTT OD/interpolated relative protein concentration) in comparison to non-treated explants. All tissues were plated in triplicates (3 wells for each condition) per plate. The values are the average of at least eight (8) plate culture replicates, including the SEM. Blue arrows show doses tested in vivo. Asterisks show *t*-test comparisons between non-treated explants and experimental groups * *p* < 0.05, ** *p* < 0.01. ^a^ All PS-based treatment samples also contain PS for a FC = 100 mg/mL.

An alternative protocol was prepared utilizing tissue explants instead of isolated cells. These explants might be a better comparison to what takes place in vivo where cells lie within a cellular or extracellular matrix milieu and where they are less exposed to the components within the surrounding fluid. Thus, 3 mm muscle explants were exposed for 72 h to the antibiotics to test their effect on their metabolic activity (Figure 5A). A correlation test failed to associate PS, K, V, and E doses to explant metabolic activity (Figure 5B,C,E,G). However, a Kruskal–Wallis assay (*p* = 0.0032) showed that the dose of kanamycin 10 μg/mL increased the tissue metabolic activity, supported by Dunn’s multiple comparisons test, but exposure to vancomycin significantly decreased it (Kruskal–Wallis test *p* = 0.0153). Even though there was no apparent correlation, various PS doses significantly increased the enzymatic activity of muscle explants (10, 30, 125, and 500 μg/mL, MW test *p* = 0.0061 and 0.0310). Moreover, when antibiotic cocktail effects were analyzed, a negative correlation for VPS treatments (Spearman r = −0.83, *p* = 0.0083) at 100 μg/mL was observed (MW test, *p* = 0.0234) (Figure 5F). The non-linear regression curve fit determines the VPS IC50 values were 175.3 μg/mL. Although, EPS doses did not show a correlation on explant viability and no IC50 was determined, a dose of 2 μg/mL erythromycin plus PS significantly decreased the metabolism of muscle explants (Figure 5H). Meanwhile, a positive correlation to KPS doses (Pearson r = 0.67, *p* = 0.05) and a significant increase at 500 μg/mL (MW test, *p* = 0.0291) was observed (Figure 5D). Consequently, kanamycin (KPS) IC50 was not determined since it appeared to increase the activity of the explant at higher concentrations. Instead, the concentration at which the activity was stimulated by 50% (EC50) was assessed using the “log(agonist) vs. response—Variable slope (four parameters)” curve fit, which approximates the EC50 of KPS to 255.4 μg/mL (Figure 5D). The results suggest that PS, kanamycin and KPS doses lower than 500 μg/mL have no antagonistic effect on muscle cells or explants, and doses of VPS lower than 50 μg/mL have no effect on explant enzymatic activity. Likewise, doses of erythromycin lower than 6 μg/mL (in a cocktail with 100 μg/mL PS) have no effect on dissociated cells, and no effect on explants treated with 4–20 μg/mL EPS (Figure 5).

### 3.7. Holothurians Gut Bacteria Growth Inhibition

**Figure 6:**
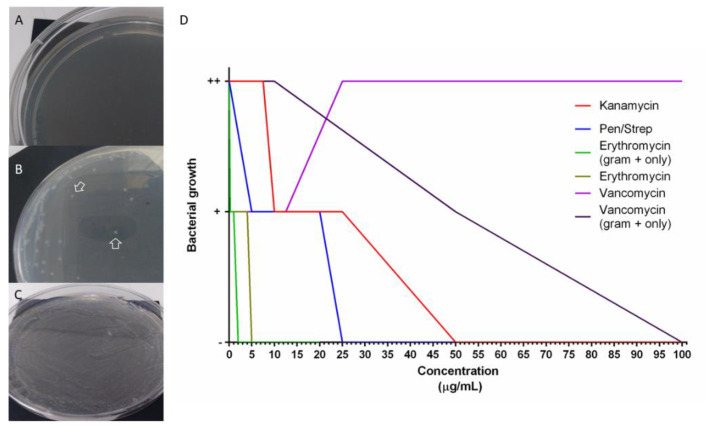
In vitro bacterial growth in antibiotic-selective media for minimum inhibitory concentration (MIC) determination. Plates with selective media incubated with holothurian gut detritus bacteria (**A**–**C**). A negative sign “-’’ was ascribed for no growth (**A**), “+” if at least one colony was seen (**B**), “++” if a full confluence lawn was seen (**C**). MIC was determined from plates with no colony formation (no bacteria growth), results from at least 4 replicates per dose were graphed (**D**).

To test the possibility that antibiotics perturb the composition of the holothurian gut microbiota we tested their effects on bacterial groups found in the holothurian gut. For this, bacteria from samples of *H. glaberrima* intestinal detritus were isolated. Gram-negative, Gram-positive, or a mixture of both were used to determine bacterial growth inhibition. MIC was performed to identify the antibiotic concentrations necessary to perturb the growth of bacteria. Treatments included penicillin/streptomycin, vancomycin, erythromycin, and kanamycin. Doses were selected based on the MTT results (Figure 5 and Figure S2) and extended to determine the concentrations that completely inhibited bacterial growth. The minimum dose necessary to clear (no bacteria growth) a sample of mixed Gram-positive and Gram-negative bacteria for kanamycin, penicillin/streptomycin, and erythromycin were 50 μg/mL, 25 μg/mL, and 5 μg/mL, respectively. Vancomycin MIC’s in cultures from mixed bacteria could not be determined since there was growth even at a dose of 325 μg/mL. Because Gram-positive bacteria are more sensitive to vancomycin, isolated Gram-positive and Gram-negative cultures were used. Again, Gram-negative bacteria did grow in all doses of vancomycin, but Gram-positive bacteria decreased at 50 μg/mL, and no bacterial growth at 100 μg/mL. The IC100 for Gram-positive inoculated in E plates, was 2 μg/mL, supporting the fact that Gram-positive bacteria are more sensitive than Gram-negative to erythromycin treatments (Figure 6).

## 4. Discussion

The work presented here shows that antibiotics alter the process of intestinal regeneration in the sea cucumber *H. glaberrima.* The possibility that this effect is caused by a direct action of antibiotics on the animal tissues versus the possibility that it might occur via changes in the microbiota is also explored. The results strongly favor the latter. 

### 4.1. The Survival Rate of Sea Cucumbers In Vivo Is Maintained after Antibiotic Treatments

The mortality in our experimental animals was independent of the supplementation with antibiotics and was probably related to the environmental changes and adaptation to the artificial sea water with decreased bacterial load. Therefore, at first glance, antibiotics do not appear to have a major effect on the organisms since the survival rate is not altered. Our results can be compared to studies in the sea cucumber *Apostichopus japonicus*, where Zhao et al. [82] explored the effect of the antibiotics erythromycin, tetracycline, and norfloxacin for 15, 30, and 45 d. They showed that while they altered the growth rate of these animals in a time-dependent manner, there was no effect on their survival [82]. This contrast between antibiotics’ effect on survival versus physiology has been seen in other organisms. For example, contrasting effects of streptomycin sulphate have been shown in some insects. In this case, the antibiotic had adverse effects (such as delayed development, reduced pupation, and late adult emergence) in *G. mellonella*, even when the survivorship was not affected [33]. Similarly, mice treated with vancomycin showed morphological villi changes in the GIT, however mortality rate remained independent to the treatment [48]. 

### 4.2. Sea Cucumber Intestinal Regeneration Is Perturbed by Antibiotic Treatments

**Figure 7:**
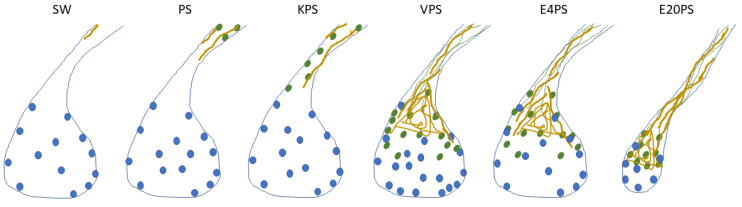
Summary of the effects of antibiotics. This scheme gathers the findings in this article on the effect of 100 µg/mL penicillin/streptomycin (PS) and PS-based cocktails including: 100 µg/mL kanamycin (KPS), 5 µg/mL vancomycin (VPS), and erythromycin 4 µg/mL (E4PS) and 20 µg/mL (E20PS) in the intestinal regeneration of sea cucumbers. Drawings were made accordingly the average results of each group to present the proliferating cells (blue dots), SLS (green ovals), muscle fibers (green lines) and collagen (yellow) localization in the regenerating rudiments and adjacent mesentery of 10-dpe animals treated with antibiotics. Briefly, administration of PS and KPS for 10-dpe only have an effect on cellular dedifferentiation while VPS, E4PS, and E20PS alter both cell dedifferentiation and ECM remodeling. Exposure to VPS also altered the cellular proliferation rate in the connective tissue of the regenerating gut, and higher doses of erythromycin (E20PS) perturbed rudiments’ growth.

In the present report, our focus was to determine whether antibiotics influence the process of intestinal regeneration of *H. glaberrima*. Exposing animals to antibiotics while undergoing intestinal regeneration showed that certain aspects of the regenerative process were altered. When using the size of the regenerating rudiment as an index of overall regenerative progress, interestingly, only animals exposed to higher concentration of erythromycin showed significant differences. However, when individual cellular events are studied, additional effects of the antibiotic treatment are detected. In the present project, PS alone (having the least dramatic effect) and KPS affected only the cell dedifferentiation process. E4PS-treated organisms presented a perturbed cell dedifferentiation and delayed ECM remodeling, and E20PS treated sea cucumber revealed the most dramatic results, also showing smaller rudiments in comparison to those of non-treated animals. Because ECM remodeling and cell dedifferentiation are processes that show similar temporal and spatial profiles, it is interesting that the drugs that delay the ECM remodeling (E20PS, E4PS, and VPS), are also characterized with the most dramatic effect (lowest *p*-values) on the degree of cell dedifferentiation compared to non-treated organisms. In addition, the proposed delay in cell dedifferentiation and in collagen depletion in VPS treated rudiments may impair the cell division pattern in regeneration intestines (Figure 7, Table 1). The elevated number of proliferating cells in the connective tissue of VPS in comparison with non-treated groups remind us of the cell proliferation indices in 7-dpe and 10-dpe respectively, giving the impression of a potential interruption of cell proliferation gradient caused by this antibiotic cocktail. Interestingly, supplementation with the higher dose of erythromycin was found to be the most prejudicial antibiotic cocktail in vivo, also inducing a significant reduction in the size of the regenerating rudiments. Thus, the effects exerted by antibiotics range between the three categories: A minor one, where only the cell dedifferentiation is perturbed; an intermediate where the ECM remodeling is also altered; and a drastic effect where the other processes, as cell proliferation or rudiment formation, are affected. An attempt to evaluate the effect of antibiotics on intestinal regeneration has previously been reported by Zhang et al. [83]. In this work, they treated *A. japonicus* sea cucumber for 3 h with 100-U/mL penicillin and 100-μg/mL streptomycin prior to evisceration and determined that antibiotic treatment increased the intestinal length and weight of regenerating animals. While these results are difficult to compare with ours, in view of the timing of the treatment and differences in quantification of results, they strongly suggest that antibiotics do have an effect on the regenerating gastrointestinal tissues.

Taken together, these results indicate that the use of antibiotics in regenerating animals perturbed the regeneration of their intestines. Our results are supported by the multiple studies that have shown the capacity of broad-spectrum antibiotics to impair intestinal histomorphology in vertebrate models. Such studies include: distorted intestinal structure with damaged villi and tight junction proteins [84,85,86], morphological changes to villi of gastrointestinal cells [48], lower levels of both SFCAs and intestinal IgA [41], and increased intestinal permeability [39]. 

### 4.3. The Metabolic Activity Remains Unaffected after Antibiotic Treatments Ex Vivo

A deeper probe into the antibiotic effects on cell and tissue metabolism was performed to elucidate if the regeneration effects could have been caused by a toxic action on holothurian tissues. The principal finding was that the concentrations used in vivo do not alter the tissue metabolic activity. In muscle explants, tested concentrations of penicillin/streptomycin and PS-based cocktails showed no negative effects, sometimes enhancing tissue activity as seen with some doses of kanamycin and KPS (Figure 5). This was not surprising, since antibiotic cocktails are commonly used as a prevention and treatment for infectious diseases [2,24,87,88,89], because of their additive or synergistic effects [90,91]. In addition, the PS-based cocktails utilized in vivo did not alter the metabolic activity of explants even when in some cases they decreased (E, EPS) or increased (K, KPS) the metabolism in dissociated cells (Figure S2 and Figure 5). The results suggest that isolated cells are more sensitive to pharmaceuticals than explants or tissues. This was also expected because of the physicochemical contrast between dissociated cells and explants. First, the dissociated cells were in suspension with more surface area directly exposed to antibiotics, versus the muscle explant where cells are found within heterogeneous compartment that may somehow impair drug penetration or where cell–cell or cell–ECM interactions stabilize cells against drug or other external effectors. Accordingly, the penetration and action of the antibiotics is dependent on the ratio of surface area to its volume [92] or tissue/serum ratio [93,94,95,96] but tissue metabolism could also affect the pharmacokinetic profiles of the antibiotics [97].

Since the tested concentrations of drugs did not drastically affect the survival of the sea cucumbers nor the metabolic activity of the animals’ tissues, we believe that the antibiotics did not have a direct effect on the intestinal regeneration processes. This led us to suspect that the tested drugs had an indirect effect by causing gut dysbiosis.

### 4.4. Antibiotics Cocktails Inhibit Gut Bacterial Populations

Our results show that, even though the doses of antibiotics used in vivo have no significant direct effect on the metabolic activity of holothurian tissues, they do hinder intestinal regeneration, potentially indicating that perturbation of the microbial community alters intestinal regeneration. To test this, we evaluated the growth of gut associated bacteria in antibiotic selecting media. In this experiment, PS was used to select against both the Gram-positive, targeted by penicillin, and the Gram-negative bacteria, targeted by streptomycin. However, due to their wide use, a large group of bacteria have gained resistance to them [98,99]. Therefore, they are used in combination with other antibiotics to create a synergistic effect and target a broad spectrum of bacteria [100]. Kanamycin, as an aminoglycoside, shares a similar action mechanism as streptomycin, targeting the Gram-negative bacteria. Vancomycin was used to target Gram-positive bacteria. This drug acts similarly to penicillin by disrupting cell wall synthesis, however there is less antibiotic resistance to vancomycin (Figure 6). The macrolide erythromycin was used to target Gram-positive bacteria as a bacterial ribosomal 50S subunit inhibitor. However, it has also been found to act against Gram-negative bacteria at high concentrations [101].

We found that the drug concentrations that inhibited gut bacteria growth ex vivo were lower than the ones used in our in vivo experiment. This suggests that the bacterial community of the intestinal digestive tract was perturbed by the antibiotic treatment and that this could have had an indirect effect on the regenerative process. An association between dysbiosis and intestinal histomorphology has also been documented in abx-treated mice [86]. These animals had a decreased SCFA production, which correlated with a decreased abundance of Firmicutes in their dysbiotic intestines. Particularly murine models treated with vancomycin presented intestinal permeability and diminished SCFA levels [39]. Additional studies associate the impaired intestinal health with induced gut microbiota dysbiosis in Nile tilapia [84,102,103,104], as well as in Atlantic salmon *Salmo salar* [105], and zebrafish *Danio rerio* [106]. Moreover, our results may indicate that the animal’s microbiota is important for the intestinal regeneration process. We found that all cocktails perturb the intestinal regeneration, but each treatment alters the intestinal regeneration in a particular array of cellular processes. Studies in the microbial ecology of sea cucumbers revealed a high relative abundance of Proteobacteria and Bacteroidetes (mostly Gram-negative bacteria), and Firmicutes (mostly Gram-positive bacteria) [107,108,109,110]. Pagán-Jiménez, M. and collogues (2019) also shows that the community is shifted toward a higher abundance of Firmicutes upon acclimatation to our lab conditions [107]. In addition, two studies have suggested that the gut microbial composition of *A. japonicus* changes during the regeneration process, and a higher diversity is observed in regenerating animals [111,112] but the fecal bacterial communities are not significantly different to non-eviscerated animals [113]. Likewise, studies in the *S. briareus* show that the intestinal microbiome of regenerating animals is more diverse at order level than the control group [110]. However, for both the gut community of *A. japonicus* and *S. briareus*, the diversity of the regenerating animals decreases at later stages of the regeneration supporting the resilience of the microbiota [110,111,112,113]. Our results may indicate that selection against Gram-positive bacteria causes larger detrimental effects in *H. glaberrima* intestinal regeneration. Therefore, some Gram-positive bacteria from the Phyla Firmicutes and Actinobacteria may be noteworthy as potential enhancers or regulators of this phenomenon, since they have the capacity of producing a variety of SCFAs, which could help control inflammation and promote epithelial repair [114,115,116,117,118]. The link between bacteria and regeneration might reside in that butyrate and other SCFAs have an inhibitory effect over histone deacetylases activity, promoting histone acetylation. Consequently, they can alter gene regulation, modulating the cell proliferation, cell differentiation, and inflammatory response of an organism, contributing to intestinal homeostasis and cancer protection [119,120,121,122,123,124,125,126].

An alternate hypothesis is that the microbial community induces varying immune responses that potentially promote tissue regeneration through gut healing [49,127,128,129]. For instance, changes in the microenvironment are observed in gut injuries, where the growth of bacteria, that potentially promotes the wound healing and regeneration of the damaged tissue, is induced [130]. Moreover, the healing of intestinal wounds induced by microbiota is often associated with cell proliferation. The anaerobic *Akkermansia* was found to induce proliferation of enterocytes adjacent to the colonic wounds of mice [130]. Another study on mice proposed that an evacuation of microbiota after hepatic surgery leads to increased Lgr5-positive cells and apoptosis in the cecal crypts, hence an impaired crypt cell homeostasis [131]. In addition, germ-free (GF) zebrafish have reduced rates of epithelial cell proliferation compared with conventionally raised specimens, suggesting that gut microbiota triggers proliferation of gut epithelium [132]. These effects have also been shown in invertebrates. For example, studies in *Drosophila* have shown that the crosstalk between the gut and the microbial community modulates stress response and promotes stem cell proliferation and epithelial regeneration [133]. Even though changes in the proliferation are often associated with luminal epithelium of antibiotic-treated intestine, in the present experiments the cell division was only altered in VPS-treated animals. This may be because, in our model, cell dedifferentiation, as well as the collagen clearance, must occur prior to the cell division to be noticed. Therefore, the effects on cell dedifferentiation and EMC remodeling must be disrupted significantly enough to affect cell proliferation in the sea cucumber.

Two other studies might prove relevant to our findings. The first is a study of *A. japonicus* juveniles where enhanced weight gain was linked to increased presence of Rhodobacterales, suggesting an effect of specific bacterial groups on holothurian development [113]. The second is a study using the planaria (*Schmidtea mediterranea*), where the presence of a bacterial group (Proteobacteria) suppressed the regenerative ability of the organism and resulted in tissue degeneration in healthy worms [134], showing a direct effect of a bacteria group on regenerative properties.

Though the mechanism of how the microbiota influences the homeostasis and regenerative response on the intestine has not yet been entirely deciphered [135], our results may provide some insights to answer it. We propose that *H. glaberrima*’s commensal bacteria in the early stages of regeneration influences primarily the cell dedifferentiation. We venture that not only the microbial target of antibiotics on the holothurian intestine, but also the dose administered will explain the extent of this perturbation. For example, the sole addition of a third antibiotic amplifies the effects of the broad-spectrum PS, but the effect of PS is intensified if the added drug is a Gram-positive targeting antibiotic and the dose is increased. These effects may alter processes dependent on cell dedifferentiation, as well as restructuration of the ECM components, epithelial to mesenchymal cellular transition, cell proliferation, and, lastly, the formation of the intestine. This might indicate that the effect of the antibiotics depends on their target mechanism and causes different results on the chronology of how the microbiota regulates intestinal regeneration. 

## 5. Conclusions

PS-based cocktails were found to alter regeneration-associated cellular events such as cell dedifferentiation, ECM remodeling, and proliferation. Interestingly, neither significant survival rate of regenerating animals, nor differential metabolism in holothurians tissues was observed upon treatments of PS-based cocktails. In contrast, antibiotics decrease the growth of enteric bacteria obtained from holothurian guts (Figure 7, Table 1). Therefore, these results may indicate that alteration of a normal microbiota might interfere with intestinal regenerative processes. Moreover, our results suggest that Gram-positive bacteria may have a pivotal role during these processes. 

Future experiments should aim at the characterization of the microbial community in regenerating sea cucumbers treated with antibiotics and their comparison to control animals. Such experiments will be crucial to test the effectiveness of antibiotics against the microbial ecology of sea cucumbers. Moreover, evaluation of differential expressed genes during antibiotic treatments, may validate the cellular events that are affected upon antibiotic treatments as well as to support our toxicity assays results.

## Figures and Tables

**Table 1:** The effects of antibiotic treatments in sea cucumbers survival rate and intestinal regeneration, on the holothurian cell and tissue activity, and in the bacteria growth inhibition.

	PS	KPS	V PS	E4PS	E20PS
Regeneration-associated processes perturbed	Cell differentiation	Cell differentiation	Cell differentiation ECM remodeling Connective tissue’s cell proliferation	Cell differentiation ECM remodeling	Cell differentiation ECM remodeling Rudiment growth
Sea cucumber’s survival rate	No effect	No effect	No effect	No effect	No effect
Disassociated cell activity	No effect	No effect	Not measured	No effect	Decreased activity
Explant activity	No effect	No effect	No effect	No effect	No effect
Bacteria growth	Inhibited	Inhibited	Inhibited	Inhibited	Inhibited

Sea cucumbers’ survival rate was not altered by the any tested dose PS KPS VPS, E4PS, and E20PS. Meanwhile the cells metabolic activity was significantly decreased by erythromycin-PS concentrations higher than 6 µg/mL, explants antibiotic activity was not altered by PS, KPS, VPS, E4PS, and E20PS doses. However, gut associated bacteria growth was inhibited even at lower concentration than the doses that animals were exposed to in vivo.

## Data Availability

The data presented in this study it is contained within this article and supplementary material.

## Supplementary Materials

The following are available online at https://www.mdpi.com/2079-7737/10/3/236/s1, Table S1: In vivo treatments design. Scheme S1: SLS and collagen classification system. Scheme S2: Representation of measurements of rudiment and proliferating cell area delimitation. Figure S1: Survival proportion after treatments with antibiotics for 10-dpe. Figure S2: In vitro toxicity assays.
