# Supplementary material for: Antibiotics Modulate Intestinal Regeneration
Source: Biology (Basel). 2021 Mar 19;10(3):236. doi: 10.3390/biology10030236 (PMC8003396; doi:10.3390/biology10030236)
Supplement: Supplementary file 1 [file biology-10-00236-s001.zip › biology-1140140-conversion supplementary.docx]

**Supplementary Material**

**Methods**

*Muscle dissection for in vivo and ex vivo assays*

Muscle tissues were obtained following the dissection protocol described by Bello, SA, et al., 2015 [76], with some modifications. Briefly, animals were anesthetized by placing them on ice for 1hr, then washed with 10% sodium hypochlorite, ethanol 70% respectively for one minute, and left in purified/autoclaved sea water. Animals were dissected, and longitudinal muscles were carefully removed. Collected tissues were transferred to a PS, neomycin, and amphotericin B antibiotic (3X abx) cold media. Upon removal, longitudinal muscles were transferred to 3X abx and left in a shaker for an hour at room temperature.

*Cell cultures* *and toxicity assays*

To obtain isolated cells, muscles were further cut into approximately 1mm^2^ pieces using scissors and washed again for 1hr in 3X abx. After centrifugation, the antibiotic solution was removed and tissues were resuspended in 5-10 ml of 0.15% collagenase (Sigma-Aldrich^R^), left shaking overnight at room temperature. Collagenase was removed by centrifugation at 25 °C for 10 min at 1600 rpm. The cells were resuspended in fresh supplemented media and washed twice to remove all traces of collagenase. A last centrifugation at 400 rpm for 10 mins was done, and the low-density phase supernatants (containing the smaller cells) were counted and plated at around 10^5^ cells per well in 100 µl of media, 10 µl of antibiotics dilutions were added.

Culture media consisted of supplemented L-15 media (13.8 g/L L-15, 12.5 g/L NaCl, 6.24 g/L glucose, 3.16 g/L MgCl_2_, 1.49g/L CaCl_2_, 300 mg/L L-glutamine, and 192 mg/L NaHCO3, 1X penicillin/streptomycin, 50 μg/ml gentamicin, 1 mM sodium pyruvate, 1% MEM non-essential amino acids, 1.75 ug/ml tocopherol, 2.5 ug/ml amphotericin B). Ten μl drug stocks with adjusted pH 7.5 were added to each well up to the final concentration, and ten μl of distilled water were added to non-treated controls, while three wells per plate were filled with media alone (blanks). The concentrations tested for PS and K included: 10, 20, 30, 40, 50, 60, 100, 125, 250, and 500 μg/mL. Because Erythromycin has been shown to work at lower concentrations, cells were treated with final concentrations of 2, 4, 6, 10, 25, 50, and 100 μg/mL. Triplicates were prepared for each dilution. Cells were incubated with the antibiotic dilutions for either 48 or 72 h at 25^o^C. The TOX1 Sigma *In Vitro* Toxicology Assay Kit (MTT based-Sigma) [78] was added to determine the metabolic activity of sea cucumber cells exposed to antibiotics. 100 µl of MTT solution (MTT reagent/ culture media) were added to each well and incubated for 3 h. As the result of metabolic activity, formazan is formed by the cleavage of the MTT tetrazolium ring after metabolic redox reactions within the cultures. Formazan production was recorded at 570 nm, (blanks’ lectures were used to eliminate media fluorescence). Additionally, readings at 690 nm were subtracted to eliminate plate background from each well. Absorbance measurements at 597 nm were obtained using the plate reader SpectraMax 360, and SoftMax Software. Results are represented as the metabolic activity rate:

| $\frac{treated absorbance}{nontreated absorbance}*100$, | (4) |
| --- | --- |

where treated absorbance and non-treated absorbance are the net absorbance of cell samples treated with antibiotics and nontreated cells, respectively. Mean results where graphed using PRISM GraphPad 6.0, and error bars shows the SEM for each treatment. In PS based cocktails assays, PS at a final concentration of 100 µg/mL was added to all dilutions except untreated controls (0 μg/mL). KPS doses included 10, 20, 30, 50, 125, 250, and 500 μg/mL and, EPS included, 2, 4, 6, 10, 25, 50, and 100 μg/mL plus a constant PS dose.

*Survival analysis*

During the *in vivo* experiments, observations of water turbidity, temperature and pH were collected before and after each water change. Also, observations of each sea cucumber were collected, including if they were attached to the aquarium surface or rocks or if they look healthy (no skin ulcers). If an animal had small (<1 cm^2^) skin ulcer, they were transferred to a new aquarium and treated separately to healthy animals. If an animal died or had extensive skin lesions (<1 cm^2^), they were removed and sacrificed, remaining animals were transferred to a new aquarium. Because surviving animals were sacrificed 10-dpe, survival rate was observed for only 10 d after treatment. Survival analysis was performed creating a Kaplan-Meier survival curve. The comparison of survival curves was done using a Log-rank (Mantel-Cox) test. Graphs and statistical analyses were performed using GraphPad Prism 6.

**Table S1:** *In vivo* treatments design

|  | Abbreviation | Treatment |
| --- | --- | --- |
| Experimental groups | PS | Penicillin-Streptomycin (100 µg/mL) ^a^ |
|  | E4PS | Penicillin-Streptomycin (100 µg/mL) + Erythromycin (4 µg/mL) ^a^ |
|  | E20PS | Penicillin-Streptomycin (100 µg/mL) + Erythromycin (20 µg/mL) ^a^ |
|  | KPS | Penicillin-Streptomycin (100 µg/mL) + Kanamycin (100 µg/mL) ^a^ |
|  | VPS | Penicillin-Streptomycin (100 µg/mL) + Vancomycin (5 µg/mL) ^a^ |
| Control group | SW | No drug added to filtered seawater (FSW) |

^a^Drug stocks were added to each aquarium (containing 1L of filtered seawater) for the final concentration specified in this table.


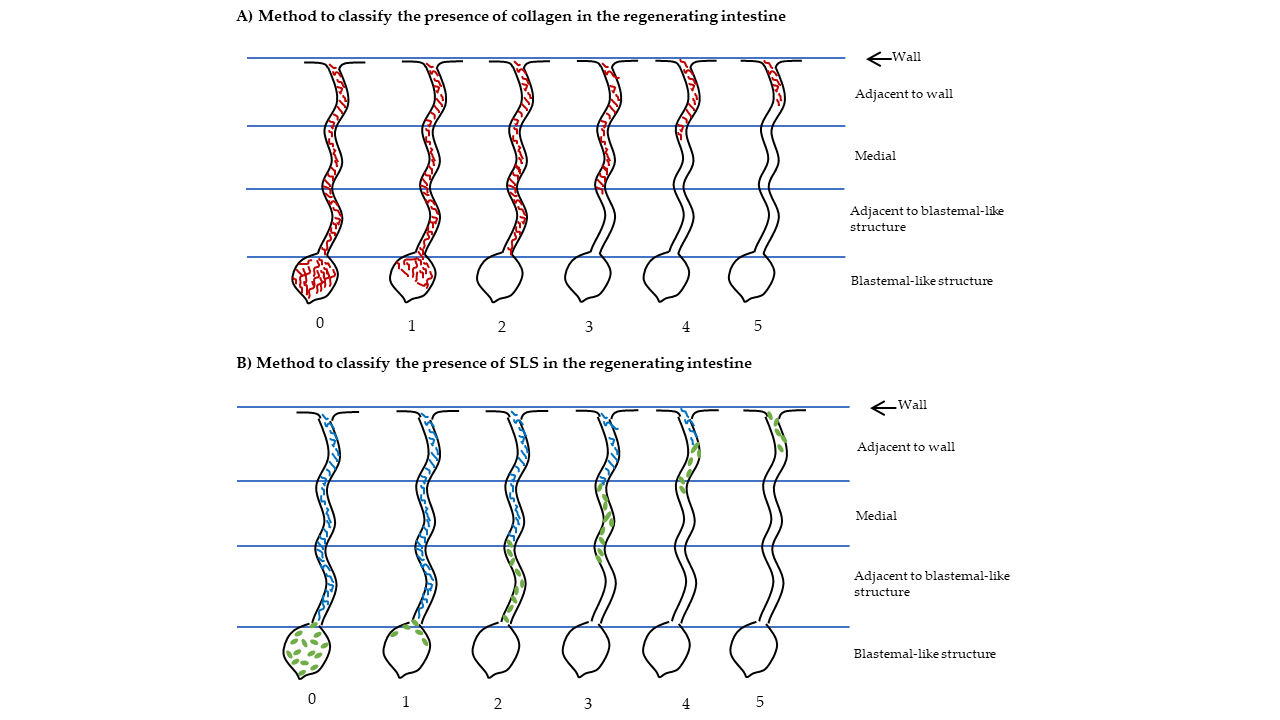


**Scheme S1:** Collagen and SLS classification system. These systems were used to quantify the presence of collagen (A) and SLSs (B) in the rudiment and mesentery. Classification ranges from 0 to 5. A classification of 0 indicates a 90%-100% of the protein in the rudiment and mesentery. A classification of 0.5 indicates 25% of the protein, and a classification of 1 indicate approximately 50% of collagen degradation in the rudiment, respectively. Classification system B was previously described by Pasten, et al., 2012 [75], with few modifications by Griselle Valentín-Tirado. Classification system A was developed by Rosa, R. based on the ECM remodeling process described by Quinoñes, et al., 2002 [71].


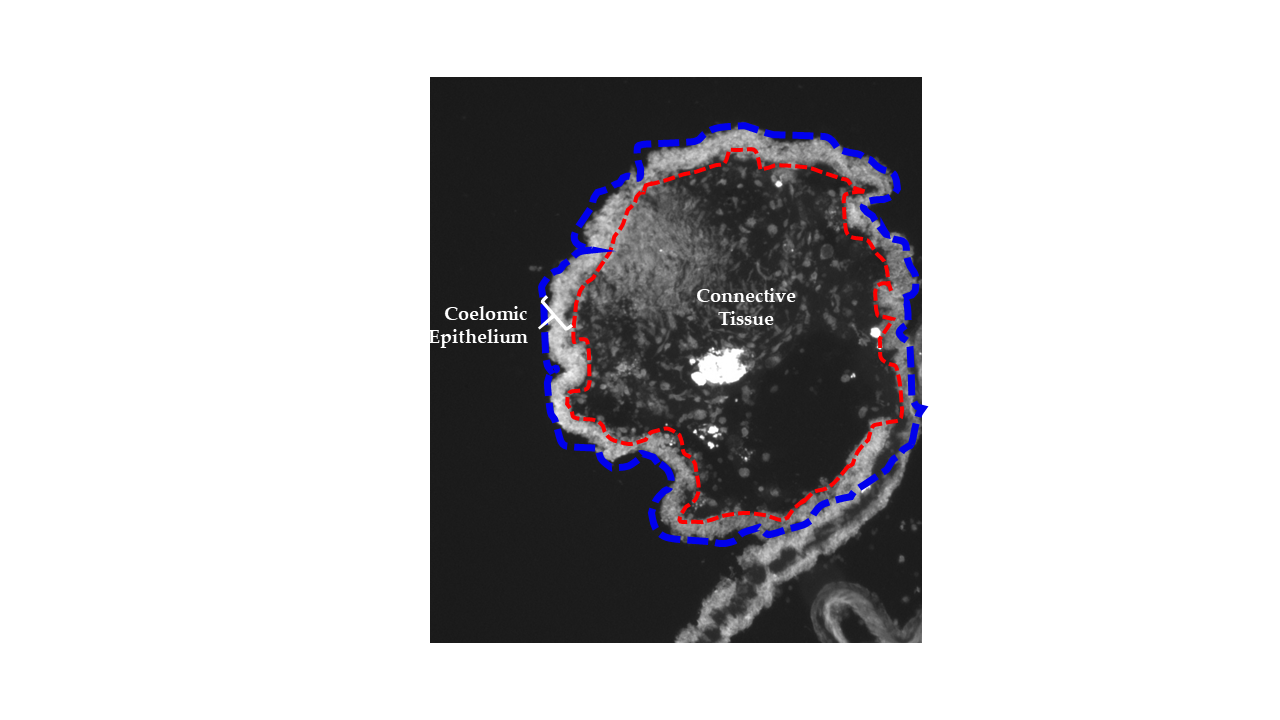
**Scheme S2:** Representation of measurements of rudiment and proliferating cell area delimitation. The blue line represents the area of the regenerated rudiment. The area delimited by the blue and red lines is the coelomic epithelium (mesothelium), and the area inside the red line represent the connective tissue (mesenchyme). The areas of the rudiments were measured, and cells were quantified both using ImageJ Software.





**Figure S1:** Survival proportion after treatments with antibiotics for 10-dpe. A Kaplan-Meier survival curve and Log-rank (Mantel-Cox) test were performed to analyze the survival rate of regeneration sea cucumbers during antibiotic treatments, surviving animals were sacrificed 10-dpe for histological analysis. “n” value represents the number of individuals at the beginning of the treatment. Results from at least nine (9) animals were graphed and analyzed using GraphPad PRISM 6.0.


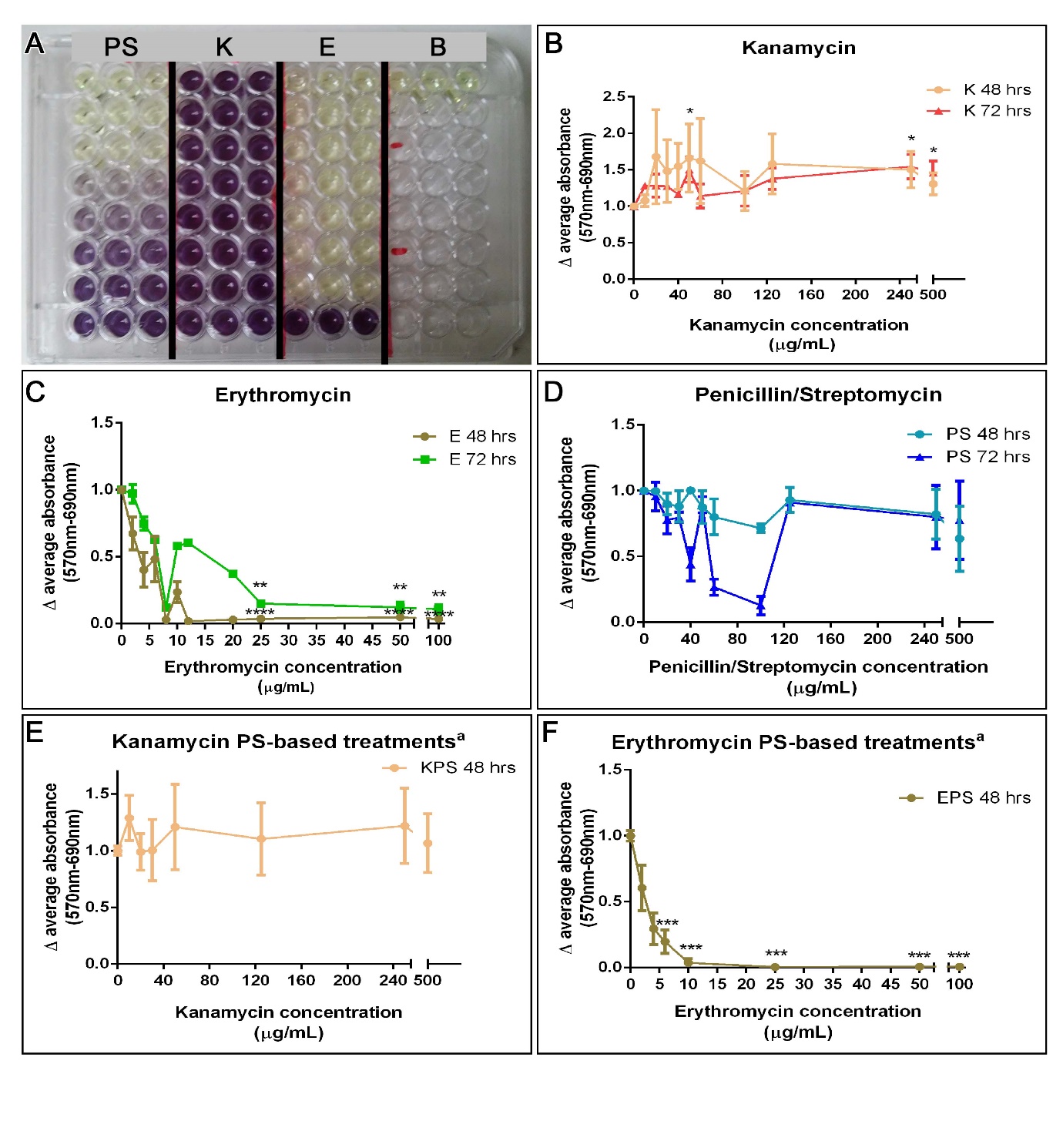
**Figure S2:** In vitro toxicity assays. A) Culture of cells treated with antibiotics (pH 7.5) after incubation with MTT reagent. B, C and D show the dose effects of K, E and PS on disassociated muscle cells after 48 hrs and 72hrs. E and F present the dose effects after 48hrs in culture when K or E, are added to PS 100 μg/mL. Cells were cultured in triplicates (3 wells for each concentration) per plate. The values are presented as the average of at least three plate culture replicates. Results are represented as rate of metabolic activity, and SEM are shown in error bars. Asterisks show t-test comparisons between non-treated cells and experimental groups * *p* < 0.05, ** *p* < 0.01, *** *p* < 0.001, **** *p* < 0.0001.

**References in Supplementary Materials** (also cited in main article)

71. Quiñones, J. L.; Rosa, R.; Ruiz, D. L.; García-Arrarás, J. E. Extracellular Matrix Remodeling and Metalloproteinase Involvement during Intestine Regeneration in the Sea Cucumber Holothuria Glaberrima. *Developmental Biology* **2002**, *250*, 181–197. <https://doi.org/10.1006/dbio.2002.0778>.

1. Pasten, C., Rosa, R., Ortiz, S., González, S., & García-Arrarás, J. E. Characterization of proteolytic activities during intestinal regeneration of the sea cucumber, Holothuria glaberrima. *The International journal of developmental biology* **2012**, *56*, 681–691. <https://doi.org/10.1387/ijdb.113473cp>.
2. Bello, S. A.; Abreu-Irizarry, R. J.; García-Arrarás, J. E. Primary Cell Cultures of Regenerating Holothurian Tissues. In *Tissue Morphogenesis*; Nelson, C. M., Ed.; Springer New York: New York, NY, 2015; Volume 1189, pp 283–297. <https://doi.org/10.1007/978-1-4939-1164-6_19>.
3. *In Vitro* Toxicology Assay Kit MTT based. Available online: h[ttp://www.sigmaaldrich.com/content/dam/sigma-aldrich/docs/Sigma/Bulletin/tox1bul.pdf](http://www.sigmaaldrich.com/content/dam/sigma-aldrich/docs/Sigma/Bulletin/tox1bul.pdf). (accessed on 25 January 2021).
