# Supplementary figures and images for: Antibiotics Modulate Intestinal Regeneration
Source: Biology (Basel). 2021 Mar 19;10(3):236. doi: 10.3390/biology10030236 (PMC8003396; doi:10.3390/biology10030236)

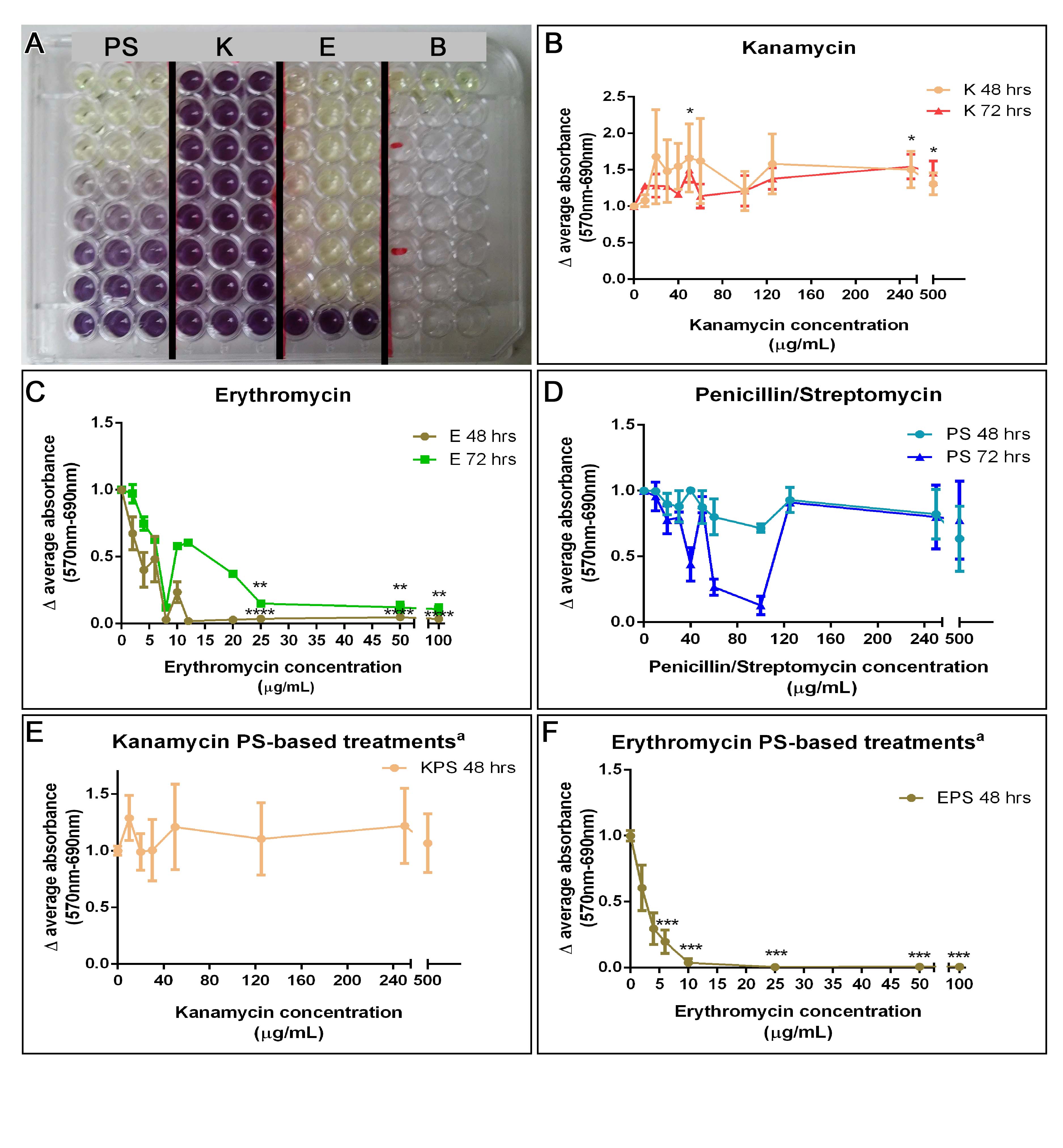

Supplement: Supplementary file 1 [file biology-10-00236-s001.zip › FigureS2.jpg]

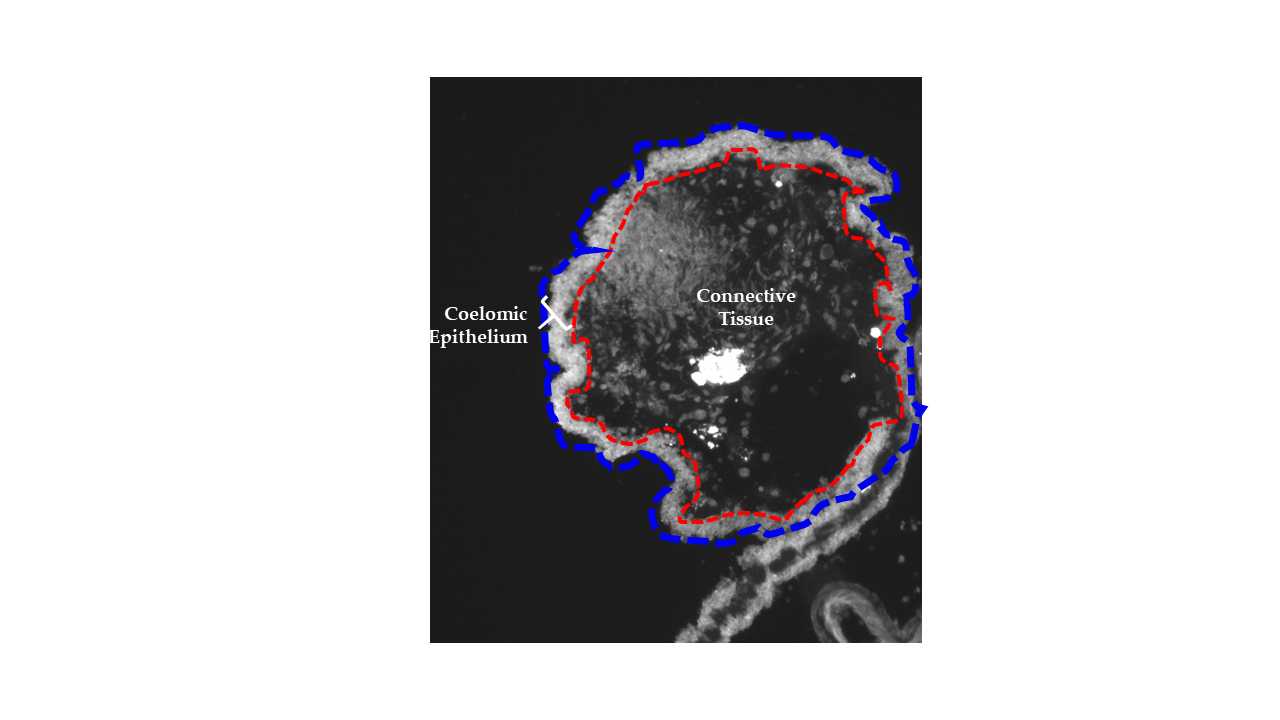

Supplement: Supplementary file 1 [file biology-10-00236-s001.zip › SchemeS2.tif]

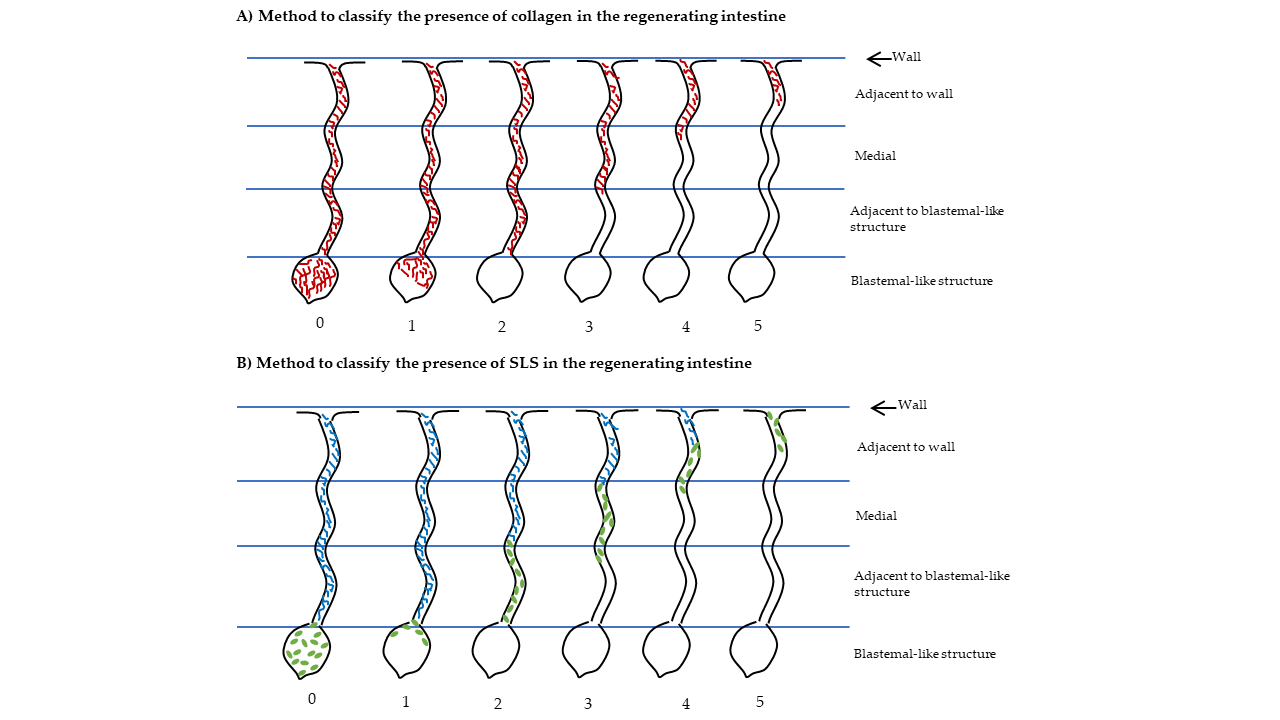

Supplement: Supplementary file 1 [file biology-10-00236-s001.zip › SchemeSI.tif]
